# Systemic cachexia and muscle–bone crosstalk drive depression-related joint remodeling and pain

**DOI:** 10.1097/JS9.0000000000004653

**Published:** 2026-02-18

**Authors:** Chen Zhao, Pengcheng Liu, Jialong Wu, Ran Duan, Weiqi Li, Jianfei Zhang, Xuzhuo Chen

**Affiliations:** a Department of Oral Surgery, Shanghai Key Laboratory of Stomatology & Shanghai Research Institute of Stomatology, National Clinical Research Center for Oral Diseases, Shanghai Ninth People’s Hospital, College of Stomatology, Shanghai Jiao Tong University School of Medicine, Shanghai, China; b Department of Orthopedics, Shanghai Key Laboratory of Orthopedics Implant, the Ninth People’s Hospital, Shanghai Jiao Tong University School of Medicine, Shanghai, China; c Department of Orthopedics, Shanghai General Hospital, Shanghai Jiao Tong University School of Medicine, Shanghai, China; d Department of Reconstructive and Plastic Surgery, Shanghai Ninth People’s Hospital, Shanghai Jiao Tong University, Shanghai, China; e State Key Laboratory of Oral Diseases, National Clinical Research Center for Oral Diseases, Chinese Academy of Medical Sciences Research Unit of Oral Carcinogenesis and Management, West China Hospital of Stomatology, Sichuan University, Chengdu, Sichuan, P.R. China; f Department of Oral & Cranio-maxillofacial Surgery, Shanghai Ninth People’s Hospital, College of Stomatology, Shanghai Jiao Tong University School of Medicine, National Clinical Research Center for Oral Diseases, Shanghai Key Laboratory of Stomatology & Shanghai Research Institute of Stomatology, Shanghai, China

**Keywords:** chronic stress model, depression-related joint pain, lipid metabolism, muscle–bone crosstalk

## Abstract

**Background:**

Joint pain is common in patients with depression, but its structural basis and molecular mechanisms remain unclear. This study aimed to investigate the underlying pathological changes and signaling pathways contributing to depression-related joint pain.

**Materials and Methods:**

Using inflammatory and chronic stress-induced mouse models of depression, we evaluated osteoclast activation, subchondral bone remodeling, and associated behavioral alterations. Molecular and genetic analyses were conducted to examine the role of the Lbp-Tlr4-Netrin-1 signaling axis and key metabolic genes including Gdf-15, LepR, and PPARγ, specifically in adipose tissue, bone marrow, and osteoclasts. Additionally, we assessed the impact of muscle degeneration on joint pathology, and conditionally deleted TGF-β1 in muscle satellite cells to determine its role in joint preservation.

**Results:**

Depression-induced joint pain was associated with increased osteoclast activity and extensive subchondral bone remodeling. The Lbp-Tlr4-Netrin-1 axis was highly upregulated in depressed subchondral bone, and its inhibition alleviated both pain-like behaviors and excessive bone resorption while mitigating depression-related weight loss. Deletion of Gdf-15, LepR, and PPARγ revealed that lipid metabolism genes significantly affect both depressive behavior and pain. Depression promoted TGF-β-mediated mesenchymal stem cell senescence and adipogenic differentiation, resembling pathological changes seen in aging and obesity. Notably, simple weight gain without metabolic correction worsened joint damage. Muscle wasting due to depression also contributed to joint pathology, and deletion of TGF-β1 in satellite cells improved joint integrity.

**Conclusions:**

Depression-related joint pain is not merely a psychological phenomenon but a complex organic disorder with defined structural and molecular underpinnings. It involves dysregulated lipid metabolism, aging-associated pathways, and multi-organ interactions between fat, muscle, bone, and the nervous system.

## Introduction

Depression in individuals with inflammatory arthritis is linked to functional deterioration and diminished response to treatment^[1,2]^. With comorbid depression, the outcomes of arthritis severity and treatment response are notably poorer^[3]^. Recently, we have encountered an increasing number of patients with depression or anxiety in outpatient clinics who complain of severe joint pain, including temporomandibular and knee joint pain. Conventional pain medications are usually ineffective in alleviating symptoms, and the number of patients exhibiting depressive symptoms owing to joint pain is increasing. This trend underscores the urgent need to better understand the mechanisms linking depression and joint pain, and to develop more effective, targeted strategies for managing pain and improving patient outcomes in this growing patient population.

Depression has a significantly high prevalence throughout an individual’s lifetime, with rates reaching 20%^[4]^. Moreover, it ranks among the top 10 risk factors for global suicide, with nearly 50 000 reported suicides per year^[5]^. The most common complaints of individuals with depression are unbearable pain and discomfort.

To address the two major questions of this study – whether depressive joint pain involves structural changes as its basis, and what mechanisms or targets drive these structural alterations – we conducted comprehensive analyses and arguments through both *in vitro* and *in vivo* experiments, and as well as clinical imaging data and survey data from scales. Our results confirmed the pathological basis of depressive joint pain, establishing it as more than a just vague psychological discomfort. The manuscript is compliant with the TITAN (Transparency In The reporting of Artificial Intelligence) guidelines 2025. Artificial intelligence (AI) was not used in the research and manuscript development^[6]^.

## Materials and methods

### Mice

C57BL/6 J mice, along with Gdf-15^KO^, LepR-flox, LepR-tdTomato, and Tgf-β1-flox mice, were purchased from Gempharmatech. LepR^f/f^ mice were crossed with Ctsk-cre mice to generate LepR^f/f^-Ctsk-cre mice^[7]^. Tgf-β1 ^flox/flox^ mice were crossed with transgenic mice expressing Pax7-cre^ERT2^ to generate inducible Tgf-β1-knockout mice, specifically in muscle satellite cells. To activate cre^ERT2^ in adult mice, tamoxifen (Sigma, T5648) at a dose of 150 mg/kg body weight was administered intraperitoneally in sunflower seed oil (Sigma, S5007) to 8-week-old mice once daily for 5 consecutive days. Littermate controls were used for all experiments. For mice subjected to a high-fat diet (HFD, 60% fat, D12492, Research Diets), male C57BL/6 J mice were fed a 60% HFD (D12492, Research Diets, Inc.) to induce obesity^[8]^. In the experiments involving aged mice, the mice were 24 months old. All mice were housed under specific pathogen-free (SPF) conditions and maintained in a controlled environment with a temperature of 23 ± 2°C, humidity between 40% and 50%, and a consistent 12-h light/dark cycle throughout the experiments. All efforts were made to reduce the sample size and minimize animal suffering.

To analyze the effect of LBP knockdown on depression-related subchondral bone remodeling, AAVs (Adeno-associated virus) (5 × 10^10^ particles per mouse) of LBP (Lipopolysaccharide Binding Protein) underexpression (GPAAV-HU6-shRNA-CMV-WPRE, Genomeditech, Shanghai, China) and vector plasmids (GPAAV-CMV-MCS-WPRE) were injected separately into the mouse tail vein at 3-day intervals^[9]^. Subsequently, mice were subjected to restraint or inflammatory cytokine stress to establish of depression-like models. In the restraint stress model, mice were placed in narrow restraint tubes for 2–3 h per day for 14 consecutive days^[10]^. Alternatively, in the inflammatory cytokine stress model, mice were administered intraperitoneally with the following concentrations: 1 μg/kg mouse TNF-α (SinoBiological), 1 μg/kg mouse IL-6 (SinoBiological), or 1 mg/kg lipopolysaccharide (LPS, Sigma)^[9]^. Von Frey, hot plate, and grip strength tests were sequentially used to assess knee joint pain in mice^[11]^. After the experiments, the mice were euthanized under anesthesia and their harvested bone tissues were fixed by immersion in 4% paraformaldehyde. Fixed tissues were prepared for subsequent micro-CT, histopathological, gene, and protein analyses. To analyze the Netrin-1 knockdown on subchondral bone remodeling and knee joint pain in the context of LBP protein injection. AAVs carrying netrin-1 knockdown sequences (GPAAV-HU6-shRNA-CMV-WPRE, Genomeditech, Shanghai, China) and vector plasmids (GPAAV-CMV-MCS-WPRE) were injected separately into the mouse tail vein, with 5 × 10^10^ particles administered for each injection. After the continuous injection of LBP (1 μg/kg, SinoBiological) protein for 14 days in WT (Wild type) and Netrin-1^−/−^ mice, the Von Frey, hot plate, and grip strength tests were sequentially employed to assess knee joint pain in mice. The method for PPARγ knockdown in the tibial marrow is similar to the one described earlier, involving the injection of an equivalent dose of AAV into the mice. The only difference is that it is directly injected into the bone marrow via the knee joint using a microsyringe. For the mice receiving direct TGF-β1 injections, 50 µL of recombinant protein factor at a concentration of 10 ng/mL was administered daily via intra-articular injection. To create the CTX-induced injury model, mice were anesthetized with isoflurane and then received an intramuscular injection of 50 µL of 8 μM cardiotoxin (CTX; MCE, HY-P1902) into the tibialis anterior muscle^[12]^. Muscles were collected for assessment 6 days post-injury^[12]^.

**HIGHLIGHTS**

- Depression-induced joint pain involves subchondral bone remodeling.
- The LBP–TLR4–Netrin-1 axis mediates bone–nerve inflammation and pain.
- Depression accelerates stem cell senescence and adipogenesis via TGF-β signaling.
- Gdf-15, LepR, and PPARγ link lipid metabolism to depression-related pain.
- Muscle–bone crosstalk drives joint pathology, reversed by TGF-β1 deletion.

All procedures were reported in accordance with the ARRIVE guidelines^[13]^. Mice were randomly assigned to experimental or control groups using a random number generator. Investigators responsible for behavioral testing, histological evaluation, and statistical analysis were blinded to group allocation throughout the study to minimize bias. To control for potential sequence effects, all behavioral and imaging assessments were performed in the same order and at consistent times of day for each animal. The unit of analysis for each experiment was defined as follows: for behavioral assays, n refers to the number of animals; for histological quantification, n refers to the number of independent animals, with three nonoverlapping fields of view randomly selected per tissue section; for cell-based assays, n indicates the number of independent biological replicates. Exclusion criteria were pre-established and included: (1) animals that exhibited severe unrelated illness or injury during the experiment; (2) samples with technical failure in staining; and (3) data points exceeding ±2 standard deviations from the group mean after verification of technical error. No animals or data points were excluded unless they met these predefined criteria.

### Cell culture

For bone marrow-derived macrophages (BMDMs), ten 6–8-week-old mice were selected for experiments. After euthanasia anddissection of the hind limbs, femurs and tibias were harvested. The bone epiphyses at both ends were removed, and the bone marrow was flushed out using a 1 mL insulin syringe. The harvested cells were cultured either in a 10 cm dish or transferred to a T75 flask. The culture medium comprised complete medium containing 30 ng/mL M-CSF, 10% fetal bovine serum (FBS), and penicillin–streptomycin. After 2 days, the cells were observed with microscope, and the medium changed for fresh medium (α-MEM was chosen as the culture medium). After an additional 2 days, numerous mononuclear macrophages derived from the bone marrow were visible under the microscope, indicating the successful extraction of BMDMs. For further induction of their differentiation into osteoclasts, BMDMs were trypsin-digested, plated, and after cell adhesion, cultured for 5–6 consecutive days in α-MEM complete medium containing 30 ng/mL M-CSF and 100 ng/mL RANKL. The observation of a significant number of ginkgo leaf-like fused cells indicates the initiation of osteoclast fusion. If fusion did not occur by day 8 or 9, the experiment was considered unsuccessful^[8]^.

Bone marrow-derived mesenchymal stem cells (BMSCs) were isolated using the same method as was used for the BMDMs. Freshly isolated single-cell suspensions were plated at a density of 5 × 10^5^ cells in 10 cm plates using BMSCs growth media (α-MEM supplemented with 10% FBS, 100 U · mL^−1^ penicillin, 100 μg · mL^−1^ streptomycin). Cells were allowed to proliferate for 3 days prior to aspirating the supernatant, rinsing with PBS three times, and changing the medium three times per week for 2 weeks. Upon reaching 80% confluence as observed under a microscope, the cells were trypsinized and seeded either on the upper or lower layer of a cell co-culture plate, as required, for the osteoclast, muscle or C2C12 co-cultures. For adipogenic differentiation, BMSCs were cultured in adipogenic media (mesenchymal stem cell adipogenic differentiation and staining kit, Meilunbio) for a specified number of days, with medium changes three times a week^[8]^.

The C2C12 cells were purchased from ProCell. Cells were maintained at 37°C under 5% CO_2_ in DMEM supplemented with 10% fetal bovine serum, 100 units/mL penicillin, and 100 µg/mL streptomycin.

### Mouse inflammatory stress model

Inflammatory cytokines were dissolved in 0.9% saline and administered intraperitoneally (i.p.). Concentrations were as follows: 1 μg/kg mouse TNF-α (SinoBiological), 1 μg/kg mouse IL-6 (SinoBiological), or 1 mg/kg lipopolysaccharide (LPS, Sigma), respectively^[9]^. Behavioral experiments and analyses to assess mouse pain were conducted after consecutive injections for 1 week, 2 weeks, and 1 month.

### Chronic restraint stress (CRS)

To induce depression-related adverse emotions, we chose a well-established and extensively validated restraint model in which mice were placed in narrow restraint tubes for 2–3 hours per day for 14 consecutive days^[14,15]^. After 14 consecutive days of restraint, behavioral experiments and analyses were conducted to assess pain in mice.

### Von frey test

Mice were placed in an enclosure with a mesh floor (30 cm long × 9 cm wide × 24 cm tall) that allowed access to the paws. The animals were given time (approximately 30 min) to acclimatize to the environment to reduce stress and ensure natural behavior during the test. After the mice were fully adapted to the environment, a series of Von Frey filaments (calibrated nylon monofilaments of varying thicknesses) were applied to the plantar surface of the paw of the animal. Each filament exerts a specific force when bent. The filament was applied until it bent slightly, indicating consistent pressure. The responses of the animals (withdrawal, licking, or shaking of the paws) were observed. The force at which each animal withdrew its paw was recorded. If there was no response, the next higher-strength hair was applied up to a maximum level that corresponded to a 15-g bending force. The test was repeated six times using different filaments to determine the threshold force that elicited a withdrawal response in 50% of the applications. This threshold indicated the mechanical pain sensitivity of the animals^[16]^.

### Hot plate test

The mice were placed in a transparent enclosure on a heated metal plate, known as a hot plate. The plate was heated to a constant temperature of 50–55°C (122–131°F). Before the test, animals were allowed to acclimatize to the testing environment to minimize stress and ensure natural behavior (approximately 30 min). The timer was started immediately after animals were placed on the hot plate. Researchers observed the animals and recorded their latency to exhibit specific pain-related behaviors such as:

Licking or shaking the hind paws

Jumping or attempting to escape

The test was stopped once the animal displayed one of these pain-related behaviors, and the time (latency) was recorded. To prevent tissue damage, a cut-off time (around 30 s) was set, after which the animal was removed from the hot plate if no response was observed.

### Grip strength test

In this test, the mouse was gently lifted by its tail and allowed to grasp a horizontal bar connected to a force transducer. It was gently pulled away from the bar until its grip was released. The force applied at the time of release was recorded as a measure of grip strength. This test was repeated six times to obtain average grip strength measurement for each animal.

### Open field test

In a dimly lit room (5–10 lux), mice were gently placed in the center of an arena measuring 40 cm × 40 cm × 40.5 cm, and allowed to explore freely for 10 minutes. A video camera was positioned directly above the arena and total locomotor activity was analyzed. Total distance traveled was used to assess locomotor ability, while the amount of time spent in the center zone served as an indicator of anxiety levels^[14]^.

### Elevated plus maze test

Mice were handled for 1–2 minutes per day over the course of 3 days prior to testing. During the experiment, each mouse was gently placed in the center of the elevated plus maze, facing the open arm. The mice were allowed to freely explore the maze for 5 minutes. A video camera was positioned directly above the arena and total locomotor activity was analyzed. The time spent in the open arm was used as an indicator of anxiety levels^[14]^.

### Sucrose preference test

Mice were individually housed and acclimated to two water bottles for a period of 2 days. Following 24 hours of food and water deprivation, the mice were presented with one bottle containing 1.5% sucrose solution and another bottle of water for a duration of 2 hours. The positions of the bottles were alternated every 30 minutes throughout the 2-hour testing period. Sucrose preference percentage was calculated based on the proportion of sucrose intake relative to the total liquid consumption^[17,18]^.

### Human research

Follow-up and voluntary questionnaire surveys were conducted among outpatients seeking treatment for temporomandibular joint discomfort. A modified PHQ-9 (Patient Health Questionnaire-9 items) depression scoring scale was used to evaluate depressive symptoms. The modified PHQ-9 included the following nine items:

1. Feeling down, depressed, irritable, or hopeless;
2. Little interest or pleasure in doing things;
3. Trouble falling asleep, staying asleep, or sleeping too much;
4. Poor appetite, weight loss, or overeating;
5. Feeling tired, or having little energy;
6. Feeling bad about yourself – or feeling that you are a failure, or that you have let yourself or your family down;
7. Trouble concentrating on things like work, reading, or watching TV;
8. Moving or speaking so slowly that other people could have noticed, or the opposite – being so fidgety or restless that you were moving around more than usual;
9. Thoughts that you would be better off dead, or of hurting yourself in some way.

With patients’ consent, their imaging data were further analyzed to determine the extent of temporomandibular joint damage in individuals exhibiting depressive symptoms. Before the survey, all patients were informed that their questionnaire results and imaging data would be used solely for scientific research purposes. Participation was entirely voluntary, and all participants signed an informed consent form. The study was approved by the proper Ethics Committee (IRB No. SH9H-2022-T43-2).

### TRAP staining

Mice were deeply anesthetized and perfused intracardially with 4% paraformaldehyde (PFA) in 0.1 M phosphate-buffered saline (PBS). The knee joints were then removed and post-fixed for 48 hours in the same fixative at 4°C. Following a 10-day demineralization process in 10% EDTA, the knee joint samples were dehydrated through an ascending gradient of ethanol (30–100%) and subsequently embedded in paraffin. Serial sections of the joints were obtained at a thickness of 5 µm and subjected to tartrate-resistant acid phosphatase (TRAP) staining using TRAP kits (Fast Red TR/Naphthol AS-MX, Sigma, St. Louis, MO). Static histomorphometric analyses of subchondral bone for osteoclast number (osteoclast number per trabecular bone surface covered by osteoclasts, Oc.S/BS) were conducted using ImageJ (NIH) based on images taken with a Leica microscope.

### Histology, immunohistochemistry, and immunofluorescence

The knee joints of the mice were fixed in 4% paraformaldehyde, decalcified, dehydrated, and embedded in paraffin. Serial sections were cut and stained with Safranin O and Fast Green (SO&FG), and Hematoxylin and Eosin (H&E) for morphological analysis, according to the manufacturer’s instructions. Cartilage destruction was graded on Safranin O-stained sections by blinded observers using the OARSI histology scoring system (grade 0–6)^[19]^. Adipose tissue samples were fixed overnight at 4 °C in 4% paraformaldehyde (PFA) and subsequently dehydrated through a series of ethanol concentrations for paraffin embedding. The paraffin-embedded tissues were then subjected to H&E staining. The analysis of adipocyte size was conducted on 8-µm-thick sections stained with H&E^[20]^. Adipose tissue axon bundles were sectioned in a transverse manner. The brains were cryo-sectioned to 5 mm thickness along the largest section. The tibialis anterior, posterior tibialis, and quadriceps femoris muscles were fixed in 2% paraformaldehyde, dehydrated, embedded in paraffin, and sectioned at a thickness of 7 µm. Necrotic muscle areas were identified through H&E staining, which highlighted regions containing necrotic myocytes, inflammatory cells, and interstitial cells. For IF staining, the sections were permeabilized with 0.5% Triton X-100, blocked with 5% BSA for 1 hour, and incubated with primary antibodies overnight at 4 °C. On the second day, after washing thrice, the sections were incubated with goat anti-rabbit IgG H&L (Alexa Fluor® 555; Abcam) or goat anti-mouse IgG H&L (Alexa Fluor® 488; Abcam) secondary antibodies (diluted 1:200) for 1 hour. For immunohistochemistry, sections were deparaffinized in xylene, followed by hydration with graded ethanol. Subsequently, they were treated with 3% H_2_O_2_ and 5% BSA and incubated overnight with specific antibodies. Next, a secondary antibody conjugated with horseradish peroxidase was added to the sections, followed by staining with 3,3’-diaminobenzidine (Sigma-Aldrich)^[21]^. Histological scoring and quantitative IF staining analyses were performed in a double-blinded manner. The antibodies used in this study are listed in the Supplemental Digital Content Table S1, available at: http://links.lww.com/JS9/G872.

### Micro-computed tomography (micro-CT)

The knee joints of the mice were dissected so that they were free from soft tissue, fixed overnight in 70% ethanol, and analyzed using high-resolution μCT (Skyscan1275, Bruker micro-CT, Kontich, Belgium). The scanner was set to a voltage of 50 kVp and a current of 200 µA, with a resolution of 5.7 µm per pixel. Sagittal images of the tibial subchondral bone were used for three-dimensional morphometric analysis. We defined a region of interest covering the entire subchondral bone compartment and used five consecutive images from the medial tibial plateau for three-dimensional reconstruction and analysis.

The three-dimensional model visualization software CTVol was used for the analysis of trabecular bone parameters in the epiphysis and the overall reconstruction of the knee joint.

### Temperature measurements

BAT temperature of mice was measured using a thermal camera (Flir). The average temperature was calculated using FLIR Tools software, with a minimum of 10 images analyzed per mouse and timepoint. All thermal image analyses were conducted in a blinded manner.

### Measurement of GDF-15

The blood was collected via cardiac puncture immediately after euthanasia and allowed to stand at room temperature for 30 minutes to facilitate clotting for serum isolation. The clotted blood was then centrifuged at 3000 g for 15 minutes at 4°C, and the serum was carefully transferred to a separate tube. GDF-15 levels were measured using an enzyme-linked immunosorbent assay (ELISA) (catalog number ab216947).

### Reverse-transcriptase quantitative PCR (RT–qPCR)

Total RNA was isolated from cultured cells using the TRIzol® reagent (Thermo Fisher Scientific, USA) following the manufacturer’s instructions. Complementary DNA (cDNA) was synthesized by reverse transcription using TaKaRa reverse transcription reagents (TaKaRa Bio Inc., Kusatsu, Shiga, Japan), and RT-qPCR was conducted using Real-Time PCR Mix (TaKaRa Bio Inc.). The expression levels of target genes were determined utilizing the 2^-ΔΔCq^ method^[22]^, with GAPDH serving as the internal reference control. The primers used were listed in Supplemental Digital Content Table S2, available at: http://links.lww.com/JS9/G872.

### Senescence β-galactosidase staining

For cells cultured in 6-well plates, the culture medium was aspirated, the cells rinsed once with PBS, and then 1 mL of β-galactosidase staining fixative added (C0602, Beyotime). The mixture was then incubated at room temperature for 15 min. The volume of the fixative and subsequent solutions was adjusted proportionally to the other types of culture plates. The fixative was removed, and the cells were washed three times with PBS for 3 min each. After the final PBS wash, 1 mL of the staining working solution was added to each well. Working solutions were prepared according to the formula provided in the reagent kit. Cells where then incubate at 37°C overnight and observed under a standard optical microscope.

### Oil red O

After induction of adipogenic differentiation, the supernatant was discarded, each well was washed with PBS 2–3 times, and 2 mL of 4% paraformaldehyde solution was added to each well for 30 min to allow for fixation. Next, the Oil Red O staining working solution was prepared by mixing saturated Oil Red O dye with distilled water in a 3:2 ratio, stirring well, and filtering through neutral filter paper. The 4% paraformaldehyde was removed, washed with PBS 2 times, and 1 mL of Oil Red O staining working solution was added per well for staining, with a staining time of 30 minutes. The Oil Red O staining working solution was removed and the cells were washed with PBS 2–3 times. Then 1 mL of PBS was added per well and the lipid-staining effects observed under an inverted microscope. Cells undergoing adipogenic differentiation exhibited orange-to-red lipid droplets, demonstrating the typical characteristics of adipogenic differentiation.

### Adipogenic induction of BMSCs and co-culture with osteoclasts

Freshly isolated single-cell suspensions were plated at a density of 5 × 10^5^ cells in 10 cm plates using BMSCs growth media (α-MEM supplemented with 10% FBS, 100 U · mL^−1^ penicillin, 100 μg · mL^−1^ streptomycin). Cells were allowed to proliferate for 3 days prior to aspirating the supernatant, rinsing with PBS three times, and changing the medium three times per week for 2 weeks. Upon reaching 80% confluence as observed under a microscope, the cells were trypsinized and seeded either on the upper or lower layer of a cell co-culture plate, as required, for the osteoclast co-cultures.

For adipogenic differentiation, BMSCs were cultured in adipogenic media (mesenchymal stem cell adipogenic differentiation and staining kit, Meilunbio) for a specified number of days, with medium changes three times a week^[8]^.

### Literature search strategy

In addition to the authors’ long-term accumulation of literature and expertise in the fields of depression, metabolism, and joint diseases, a systematic literature search was conducted to ensure comprehensive background coverage and identify research gaps relevant to this study. The search was performed across PubMed and Web of Science databases.

The following combinations of keywords were used:

“depression-related joint pain” OR “chronic stress model” OR “osteoclast activation” OR “subchondral bone remodeling” OR “lipid metabolism” OR “GDF-15” OR “PPARγ” OR “TGF-β signaling” OR “muscle–bone crosstalk.”

Reference lists of key review articles and primary studies were also manually screened to identify additional relevant publications. Studies were included if they addressed molecular or structural mechanisms linking depression, metabolism, and musculoskeletal pathology. No language restrictions were applied.

This comprehensive literature search provided a conceptual framework for the experimental design, guided the selection of molecular pathways (e.g., Lbp–Tlr4–Netrin-1), and supported the interpretation of our findings on the systemic mechanisms underlying depression-related joint pain.

### Statistics

This study did not use statistical methods to determine the experimental sample size in advance, but referred to previous related studies. The sample sizes used in each set of experiments in this study are displayed in the figures and legends. All data are expressed as mean ± standard deviation (s.d.). The number of mice used for each genotype is indicated in the figure legends. Comparisons between two groups were analyzed using a two-tailed unpaired Student’s *t*-test. For comparisons involving multiple groups, ANOVA followed by a post hoc Tukey test was employed as described in the figure legends. A *P*-value of <0.05 was considered statistically significant. All statistical analyses were conducted using GraphPad Prism 8.0.

## Results

### Depressive joint pain has an underlying pathological structure in the joint

**Figure 1.**
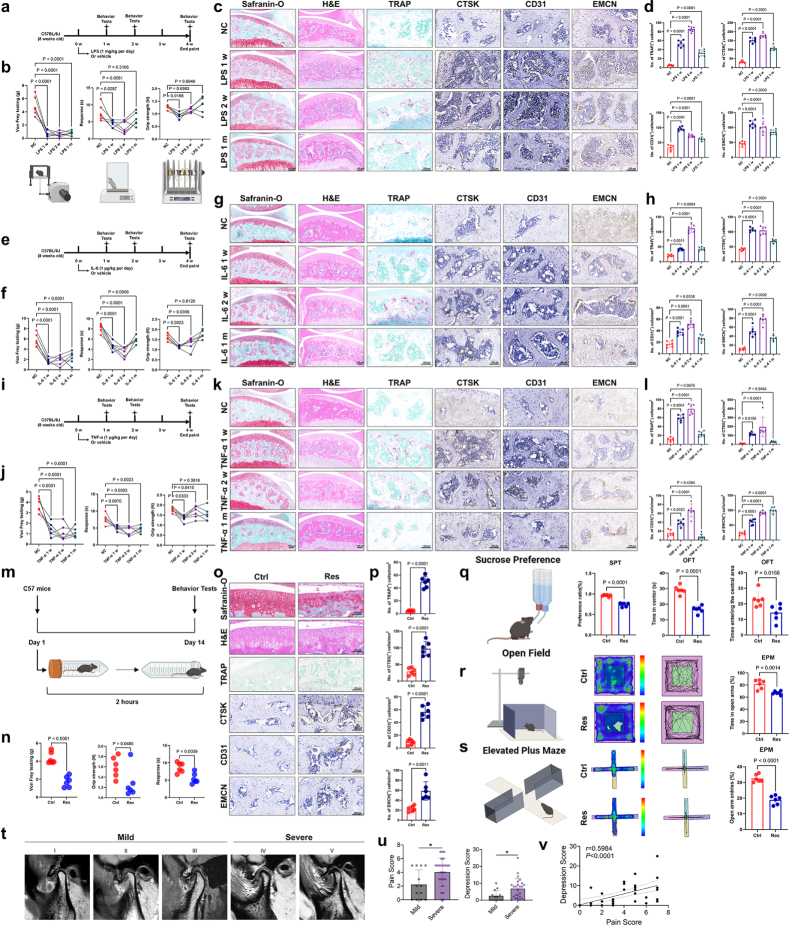
Depressive joint pain has an underlying pathological structure in the joint. (a) Schematic diagram of the injection concentration and frequency of LPS, as well as the relevant time points for behavioral testing. (b) Von Frey assay (left), hot plate test (center), and grip strength test (right) were conducted 1 week, 2 weeks, or 4 weeks after LPS injection (n = 6). (c, d) Safranin O/Fast Green, HE, and TRAP staining images of tibial subchondral bone at 1, 2, and 4 weeks after LPS injection. Immunohistochemical analysis and quantification of the number of CTSK^+^, CD31^+^, and EMCN^+^ (all stained brown in the images) cells (per mm²) in the subchondral bone of mice 1, 2, and 4 weeks after LPS injection (n = 6). Scale bars, 100 μm. (e) Schematic diagram of the injection concentration and frequency of IL-6, as well as the relevant time points for behavioral testing. (f) Von Frey assay (left), hot plate test (center), and grip strength test (right) were conducted 1 week, 2 weeks, or 4 weeks after IL-6 injection (n = 6). (g, h) Safranin O/Fast Green, HE, and TRAP staining images of tibial subchondral bone at 1, 2, and 4 weeks after IL-6 injection. Immunohistochemical analysis and quantification of the number of CTSK^+^, CD31^+^, and EMCN^+^ (all stained brown in the images) cells (per mm²) in the subchondral bone of mice 1, 2, and 4 weeks after IL-6 injection (n = 6). Scale bars, 100 μm. (i) Schematic diagram of the injection concentration and frequency of TNF-α, as well as the relevant time points for behavioral testing. (j) Von Frey assay (left), hot plate test (center), and grip strength test (right) were conducted 1 week, 2 weeks, or 4 weeks after TNF-α injection (n = 6). (k, l) Safranin O/Fast Green, HE, and TRAP staining images of tibial subchondral bone at 1, 2, and 4 weeks after TNF-α injection. Immunohistochemical analysis and quantification of the number of CTSK^+^, CD31^+^, and EMCN^+^ (all stained brown in the images) cells (per mm²) in the subchondral bone of mice 1, 2, and 4 weeks after TNF-α injection (n = 6). Scale bars, 100 μm. (m) Behavioral timeline of restraint (Res), CRS induction, and depression-like phenotype measurement. (n) Von Frey assay (left), hot plate test (right), and grip strength test (middle) were conducted 2 weeks after CRS induction (n = 6). (o, p) Safranin O/Fast Green, HE, and TRAP staining images of tibial subchondral bone at 2 weeks after CRS induction. Immunohistochemical analysis of CTSK^+^, CD31^+^, and EMCN^+^ (all stained brown in the images) cells (per mm²) in the subchondral bone of mice 2 weeks after CRS induction. Quantitative analysis of TRAP-positive cells in subchondral bone marrow (n = 6). Quantification of the number of CTSK^+^, CD31^+^, and EMCN^+^ (all stained brown in the images) cells (per mm²) in the subchondral bone of mice 2 weeks after CRS induction (n = 6). Scale bars, 100 μm. (q) Sucrose preference ratio of CRS mice (n = 6). (r) Representative locomotion traces and quantitative analysis of the OFT data (n = 6). (s) Representative locomotion traces and quantitative analysis of the EPM data (n = 6). (t) Representative TMJ MRI of patients in different TMJOA stages. Patients were divided into mild and severe according to the Wilkes classification. Mild OA includes stage I-III, and severe OA includes stages IV and V. (u) The level of pain score and depression score of patients with mild and severe OA (means ± SD, unpaired two-tailed t test). (v) Pearson correlation analysis between pain score and depression score (Pearson analysis). All data are shown as means ± SD. One way ANOVA for (b, d, f, h, j, l) with multiple comparisons. Student’s t test applied for (n, p, q, r, s, u). Pearson correlation analysis between pain score and depression score applied for (v).

First, we established a mouse model of depression using LPS. As observed in our clinical practice, depressed mice similarly exhibited heightened levels of lower limb pain (Fig. 1a, b). Similarly, this change is not just a psychological effect; the subchondral bonestructure of the knee joint also underwent corresponding changes. After 2 weeks of continuous LPS injection, the remodeling of the subchondral bone and the differentiation of osteoclasts in the knee joint of mice reached a peak compared with those of the control group, showing an evident loose and damaged phenotype (Fig. 1c, d). As the primary protease within osteoclasts, CTSK (Cathepsin K) exhibited peak expression trend in the subchondral bone during the second week, similar to that observed with TRAP staining (Fig. 1c, d). Increased subchondral bone angiogenesis and blood vessel branching are clinical features of osteoarthritis (OA). Our data indicate that during the process of depression model, enhanced invasion of type H blood vessels corresponds to increased subchondral bone remodeling and active osteoclast differentiation (Fig. 1c, d). This provides additional evidence linking depression to joint pain and remodeling. Subsequently, we used two other classic inflammatory cytokines, IL-6 and TNF-α (Fig. 1e, 1i). Consistent with the behavioral experiment results and histopathological findings in the LPS injection group, using inflammatory cytokines elevated pain levels in mice and promoted subchondral bone remodeling, active osteoclast differentiation, and increased levels of vascular invasion (Fig. 1f-l). Thus, we believe that joint pain in patients with depression is not merely a psychological manifestation or self-doubt but rather a disease with tangible organic pathological changes.

The mouse restraint model is commonly used to study stress and depression^[14]^. However, no study has been conducted on the structural changes in knee joints caused by this model. After being restrained for 2 hours each day continuously for 14 days, we assessed pain levels in the mice (Fig. 1 m). Consistent with the results from the inflammatory cytokine group (Fig. 1a-l), mice subjected to restraint also exhibited an increase in pain sensitivity and a decrease in the willingness to use force (Fig. 1 n). The results of histological staining initially indicated significant abnormal remodeling of the subchondral bone and upregulation of osteoclast differentiation in restrained mice (Fig. 1o, p). Immunohistochemistry further suggested that these structural changes were accompanied by increased expression of osteoclast differentiation proteases and substantial enhancement of vascular invasion within the subchondral bone (Fig. 1o, p). In the CRS model established after the confinement of mice, we further validated whether the disruption of this joint environment was indeed caused by depression. This included the anhedonia-like phenotypes measured by the sucrose preference test (SPT) (Fig. 1q), as well as the behavioral despair assessed by the open field test (OFT) (Fig. 1r) and plus-maze experiment (EPM) (Fig. 1s). Our experimental results demonstrated that restraint-induced depression and inflammatory cytokine injection caused elevated pain sensitivity and decreased force usage in mice. Furthermore, restraint alone caused significant structural changes in the subchondral bone, characterized by abnormal remodeling, increased osteoclast differentiation, upregulation of osteoclast differentiation proteases, and enhanced vascular invasion. These findings suggest a direct link between depression and knee joint pain, highlighting the multifaceted nature of depression-induced musculoskeletal alterations.

Finally, to correlate with the data from the mouse experiments, we leveraged the advantage of our research unit being the largest temporomandibular joint disease center in Asia to collect clinical and imaging data from patients with depression accompanied by temporomandibular joint pain. Following the specified inclusion criteria, 38 patients with temporomandibular joint osteoarthritis (TMJOA) were recruited and categorized into two stages (mild and severe) based on the Wilkes classification system, a well-recognized classification system for TMJOA (Fig. 1 t). As anticipated, the pain levels were notably higher in OA patients compared to relatively healthier individuals. Similarly, the levels of depression exhibited a marked increase in patients with severe OA as opposed to those with mild OA (Fig. 1 u). These findings indicate a potential positive association between pain, depression, and the progression of OA. Moreover, analysis using Pearson correlation revealed a positive link between pain scores and depression scores (Fig. 1 v). Collectively, these clinical findings suggest a positive correlation between depression and pain in patients with OA.

### Dopamine supplementation reverses stress-induced subchondral bone abnormalities and pain

**Figure 2.**
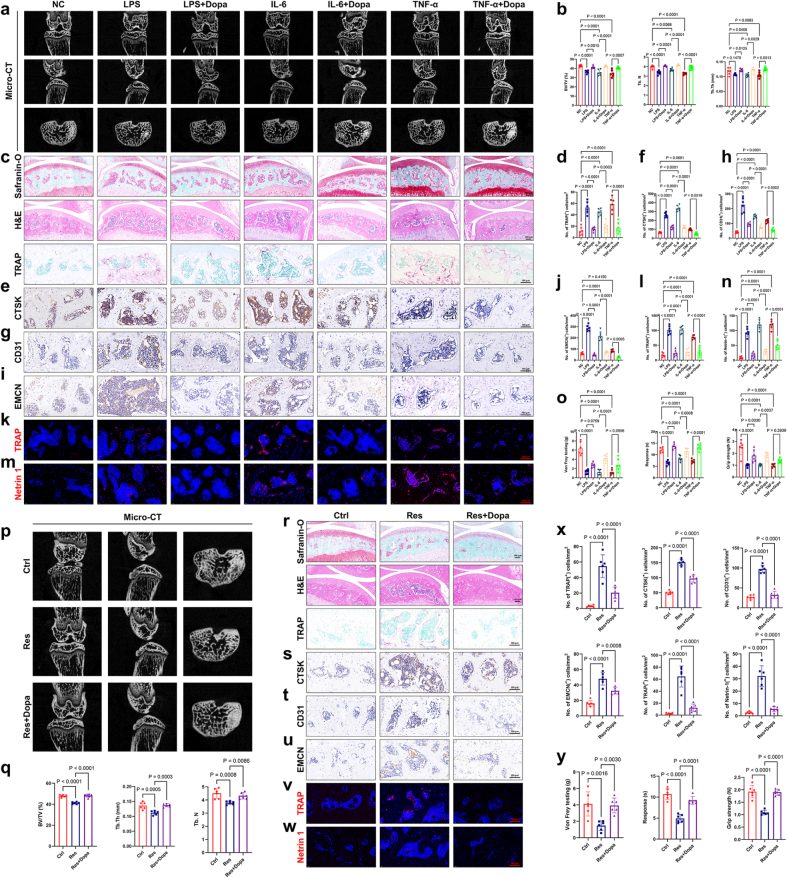
Synchronous dopamine supplementation improves subchondral bone remodeling and pain in stress-induced depression. (a) Representative µCT images of transverse, coronal, and sagittal views of the tibial subchondral bone of mice. Mice were administered intraperitoneal injections of 1 mg/kg LPS, 1 μg/kg IL-6, and 1 μg/kg TNF-α for two consecutive weeks, with concurrent injections of 10 mg/kg dopamine. (b) A quantitative analysis of structural parameters of subchondral bone (n = 6). (c) Safranin O/Fast Green, HE, and TRAP staining images of tibial subchondral bone. Scale bars, 100 μm. (d) Quantitative analysis of TRAP-positive cells in subchondral bone marrow (n = 6). (e-n) Immunohistochemical and immunofluorescence analysis and quantification of the number of CTSK^+^ (e), CD31^+^ (g), EMCN^+^ (i), TRAP^+^ (k), and Netrin 1^+^ (m) (immunohistochemically stained brown in the images, immunofluorescently stained red in the images) cells (per mm²) in the subchondral bone of mice. The grouping and treatment methods are the same as in (a) (n = 6). Scale bars, 100 μm. (o) Von Frey assay, hot plate test, and grip strength test were conducted at the end of the second week (n = 6). (p) (q) Representative µCT images of transverse, coronal, and sagittal views of the tibial subchondral bone, along with quantitative analysis of structural parameters (q) (n = 6). (r) Safranin O/Fast Green, HE, and TRAP staining images of tibial subchondral bone. Scale bars, 100 μm. And quantitative analysis of TRAP-positive cells in subchondral bone marrow (x) (n = 6). (s-x) Immunohistochemical and immunofluorescence analysis and quantification of the number of CTSK^+^ (s), CD31^+^ (t), EMCN^+^ (u), TRAP^+^ (v), and Netrin 1^+^ (w) (immunohistochemically stained brown in the images, immunofluorescently stained red in the images) cells (per mm²) in the subchondral bone of mice (n = 6). Scale bars, 100 μm. (y) Von Frey assay, hot plate test, and grip strength test were conducted 2 weeks after CRS induction.All data are shown as means ± SD. One way ANOVA with multiple comparisons was used to test for significance.

In this part of the study, we used three-dimensional microcomputed tomography (micro-CT) to visually analyze the remodeling of subchondral bone in mice (Fig. 2a). The mass of the subchondral bone decreased 2 weeks after inflammatory cytokine injection, with the bone volume to total volume (BV/TV) ratio ranging from 43.44% to 32.08%. However, the loss of bone mass and structural remodeling were improved and rescued through simultaneous dopamine supplementation (Fig. 2a, b). Changes in the subchondral trabecular number (Tb.N) and trabecular thickness (Tb.Th) were consistent with this trend (Fig. 2b, right). Similarly, the number of TRAP^+^ cells increased rapidly during the 2 weeks following inflammatory cytokine injection and decreased after dopamine supplementation (Fig. 2c, d). Immunohistochemistry results showed that the number of CTSK-positive cells was consistent with the number of TRAP^+^ cells (Fig. 2e, f). Studies have shown that dopamine is crucial in regulating the function of hematopoietic stem and progenitor cells (HSPCs) in the bone marrow^[23]^. Blocking dopamine synthesis or pharmacologically or genetically inactivating the D2 subfamily of dopamine receptors caused decreased HSPC frequency, inhibited proliferation, and reduced bone marrow transplantation efficiency. Therefore, we speculated that direct dopamine supplementation would also have an ameliorative effect on the abnormal vascular invasion of the subchondral bone. Dopamine supplementation inhibited osteoclast differentiation, and improved vascular invasion, which are critical factors in joint pain (Fig. 2g-j). Immunofluorescence experiments further confirmed the changes in osteoclast differentiation trends in the subchondral bone (Fig. 2k, l). A decrease in Netrin-1 is likely directly linked to reduced osteoclast differentiation and pain relief^[16,24]^. Our results indicate a significant upregulation of netrin-1 expression in the subchondral bone in the depression model. Dopamine supplementation, meanwhile, not only alleviated subchondral bone remodeling and osteoclast differentiation but also suppressed its aberrant overexpression, prompting further investigation into the pivotal regulatory role of this protein in depression-related pain (Fig. 2m, n). Finally, Dopamine supplementation indeed alleviated depression-like joint pain, as evidenced by improved pain tolerance, increased endurance, and enhanced muscle strength (Fig. 2o).

Compared with the group subjected to restraint alone, pre-supplementation with dopamine caused increased pain tolerance and willingness to use muscle strength (Fig. 2y). The results from micro-CT also indicated the beneficial effects of timely dopamine supplementation on subchondral bone remodeling and bone mass improvement (Fig. 2p, q), as well as its inhibitory effect on excessive osteoclast differentiation in the subchondral bone (Fig. 2r, x). The immunohistochemistry and immunofluorescence results were consistent with the changes observed in the inflammatory cytokine group (Fig. 2s-x). These results further suggest that joint pain and structural changes caused by depression can be improved through treatments targeting depression.

### The LBP–TLR4–netrin-1 axis mediates depression-induced subchondral bone damage

LBP expression was increased in individuals with depression and by diverse stress challenges in mice^[9]^. LBP has also been found to be upregulated in the blood of depressed patients^[25,26]^. First, in the brain tissue, CRS caused abnormal overexpression of LBP. This excessive upregulation was alleviated after dopamine supplementation (Extended Data Fig. 1a). Immunofluorescence experiments using brain tissue also confirmed the downregulation of DRD2 expression in mice caused by depression and the improvement effect of dopamine activation (Extended Data Fig. 1a).

The results showed significant upregulation of LBP protein and high expression of TLR4 in the subchondral bone of depressed mice (Extended Data Fig. 1b, c). Additionally, consistent with the depression induced using restraint, inflammatory cytokines also caused the overexpression of TLR4 in the subchondral bone and an upregulation of TRAP protein, suggesting that the excessive differentiation of subchondral osteoclasts induced by depression is possibly owing to the overexpression of TLR4 caused by the excessive upregulation of LBP (Extended Data Fig. 1d-f).

Then, LBP knockdown using AAV (Adeno-Associated Virus) was administered intravenously at 3-day intervals, with the first injection conducted prior to the induction of mouse restraint or inflammatory cytokine injection. The effects of LBP deficiency on depression-like subchondral bone remodeling were further investigated. As expected, in Lbp-knockdown (Lbp^-/-^) mice, no evidence of subchondral bone rarefaction or abnormal remodeling was observed following either confinement or consecutive injections of inflammatory cytokines (Extended Data Fig. 1 g, n-p). Immunofluorescence experiments revealed that in Lbp^-/-^ mice, no osteoclast overactivation was induced by depression, and the synchronous overexpression of TLR4 protein was observed (Extended Data Fig. 1 h, q, r). By revalidating the expression levels of the LBP protein in the subchondral bone, we confirmed the success of LBP knockdown and the reliance of the improvement in depression-related osteoclast hyperactivity and remodeling on LBP knockdown (Extended Data Fig. 1i, s). Next, through immunohistochemical experiments targeting the osteoclast differentiation marker CTSK (Extended Data Fig. 1 j, t), vascular invasion markers CD31 (Extended Data Fig. 1k, u), and EMCN (Extended Data Fig. 1 l, v), we confirmed significant improvements in depression-induced osteoclast differentiation and depression-related abnormal vascular invasion in Lbp^-/-^ mice. Finally, immunofluorescence experiments targeting Netrin-1 also indicated a crucial regulatory role of LBP on Netrin-1, revealing the significant regulatory potential of LBP on joint pain in depressed mice (Extended Data Fig. 1 m, w). These results demonstrate the significant regulatory role of LBP in depression-related pain and abnormal subchondral bone remodeling.

### Reversal of subchondral bone remodeling and pain induced by direct injection of LBP protein via netrin-1 knockdown

We initially generated Netrin-1^-/-^ mice using the same method. Subsequently, we administered LBP protein directly via intraperitoneal injection to WT and Netrin-1^-/-^ mice without restraint or the use of inflammatory factors. The imaging data revealed that the direct injection of LBP protein was sufficient to induce subchondral bone resorption and abnormal bone remodeling. Notably, this osteolytic effect was no longer observed in Netrin-1^-/-^ mice (Extended Data Fig. 2a, i-k). Furthermore, we confirmed the upregulation of Netrin-1 in the subchondral bone following the direct injection of LBP protein. While demonstrating the efficacy of Netrin-1 knockout, this further suggests that LBP may exert pain-modulating effects by regulating the expression of Netrin-1 (Extended Data Fig. 2b, l). Subsequent experiments involving Safranin O-Fast Green and TRAP stainings, and immunohistochemistry targeting CTSK were conducted to validate the conclusions drawn from Extended Data Figure 2a. These results confirmed the significant promotion of osteoclast differentiation by LBP protein, which was notably reversed in Netrin-1 knockout (Extended Data Fig. 2c-e, m-n). Markers of vascular invasion, another crucial key factor promoting joint pain, also exhibited elevated expression after LBP injection, which was absent in Netrin-1^-/-^ mice (Extended Data Fig. 2f, g, o, and p). Our results also indicate that the injection of LBP protein similarly induces the excessive invasion of CGRP-positive nerves in the subchondral bone, with the silence of Netrin-1 laying the foundation for pain relief (Extended Data Fig. 2h, q). Finally, we performed behavioral experiments to validate whether LBP protein could directly induce mouse joint pain and the ameliorating effect of Netrin-1. The results indicated that LBP worsened pain tolerance in mice, similar to the depression model, whereas this pain-promoting effect was not observed in Netrin-1^-/-^ mice (Extended Data Fig. 2r-t). Together, these findings indicate that depression-induced joint pain is directly caused by LBP-mediated regulation of Netrin-1 expression.

### Depression drives a joint-specific aging phenotype despite systemic cachexia

**Figure 3.**
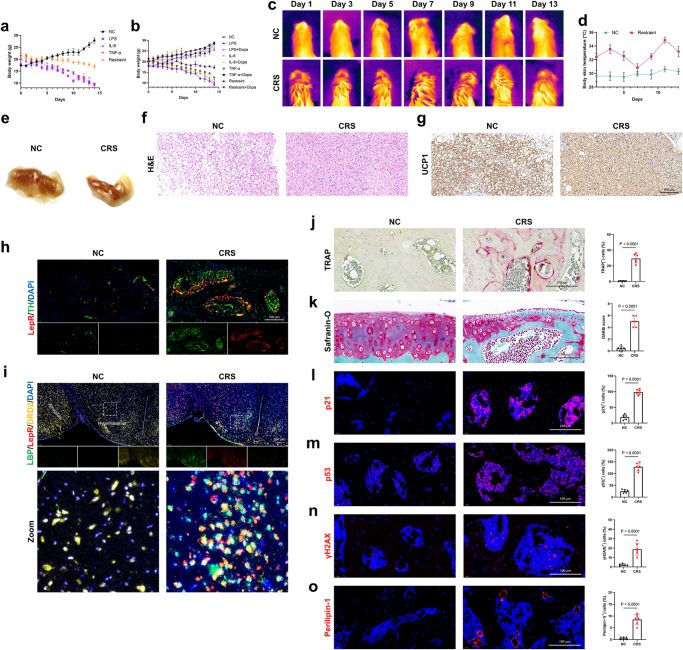
Depressed mice show mental state-linked weight changes and increased bone adipogenesis, similar to obese and aged mice. (a, b) Changes in body weight over two weeks for the NC group, inflammatory cytokines, and restrained mice, with or without the administration of dopamine (n = 6). (c, d) Thermal imaging (c) and quantification (d) at room temperature (21°C) (n=6). (e) Representative photographs of brown adipose tissue (BAT) from the control and depressed mice. (f) Representative H&E-stained sections of BAT from the control and depressed mice. Scale bars, 100 μm. (g) Representative immunohistochemistry images of BAT from the control and depressed mice. Scale bars, 100 μm. (h) Sympathetic axon bundles isolated from subcutaneous brown adipose tissue (BAT) are enclosed by a barrier of LepR^+^ cells. Tyrosine hydroxylase (TH, green) and LepR (red) staining are shown. Scale bars, 100 μm. (i) Expression of LBP (green), LepR (red), and DRD2 (yellow) in the hypothalamus. Scale bars, 100 μm. (j) Representative TRAP-stained images of subchondral bone from control and depressed mice, along with quantitative analysis (n=6). Scale bars, 100 μm. (k) Safranin O/Fast Green (SO/FG) staining of knee articular cartilage and the OARSI grade of knee articular cartilage from control and depressed mice (n=6). Scale bars, 100 μm. (l-o) Immunofluorescence staining of p21 (l), p53 (m), γH2AX (n), and Perilipin-1 (o) in the subchondral bone of joints from control and depressed mice. The percentage of positive cells in the bone marrow was quantified (n=6). Scale bars, 100 μm. All data are shown as means ± SD. One way ANOVA for (a, b) with multiple comparisons. Student’s t test applied for (d, j, k, l, m, n, o).

During the modeling process of the mouse depression model, we had to take note of a significant cachexia trend in depressed mice, strongly correlated with the duration of the modeling process (Fig. 3a). This cachexia change was reversed when dopamine supplementation was used to alleviate the progression of depression (Fig. 3b). Therefore, we speculated whether the depression-induced cachexia might be linked to depression-related joint pain and subchondral bone changes.

The thermographic imaging of the dorsal adipose tissue in depressed mice showed a marked increase in heat production and energy expenditure compared to the control group (Fig. 3c, d). This finding was consistent with histological analysis, which indicated that BAT adipocytes in the depression group were smaller and contained more multilocular lipid droplets (Fig. 3e, f). Immunostaining confirmed elevated Ucp1 levels in the BAT of the depression group compared to the normal controls (Fig. 3g), suggesting that the weight loss induced by depression was aligned with changes in dorsal fat tissue. Additionally, the sympathetic nerves innervating the dorsal BAT exhibited upregulated expression of leptin receptors (Fig. 3h). The body temperature regulation function is primarily located in the hypothalamus of the brain. We discovered that the hypothalamus exhibited high expression of LBP protein and low expression of DRD2, along with elevated leptin receptor signaling (Fig. 3i). This finding aligns with the increased energy expenditure observed in adipose tissue thermogenesis.

The above results suggest that depression-induced cachexia is not the direct cause of depressive joint damage but may be related to bone-specific factors resulting from the cachexia associated with depression. In this study, we also observed that despite depression leading to cachexia in mice, their bones exhibited increased aging and adipogenic differentiation (Fig. 3j-o). These pathological changes resemble those seen in the joints of obese and aging mice, suggesting that depression may disrupt the metabolic regulation of joints in a similar manner (Extended Data Fig. 3).

### GDF-15 deficiency dissociates systemic cachexia from local joint destruction

**Figure 4.**
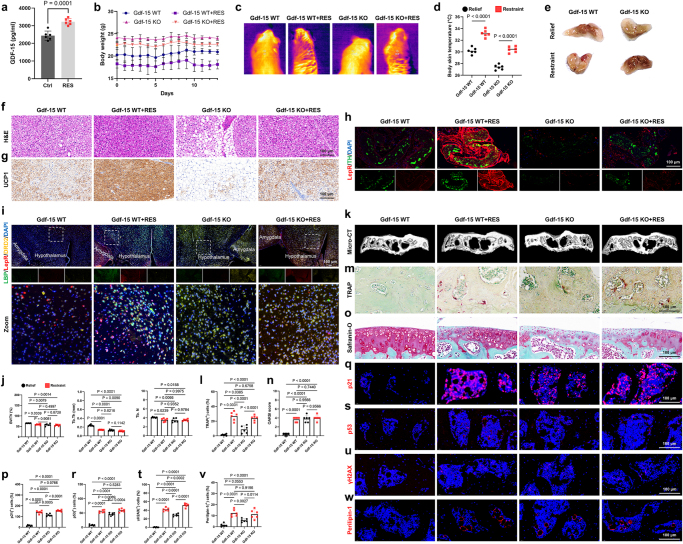
GDF15 deficiency on depressive cachexia and joint destruction. (a) Levels of GDF-15 in the plasma of control and CRS model mice (n = 6). (b) Body weight of control and restrained mice in WT and Gdf-15 KO groups (n=6). (c, d) Thermal imaging (c) and quantification (d) at room temperature (21°C) (n=6). (e) Representative photographs of BAT from WT and Gdf-15 KO mice. (f) Representative H&E-stained sections of BAT from WT and Gdf-15 KO mice. Scale bars, 100 μm. (g) Representative immunohistochemistry images of BAT sections from WT and Gdf-15 KO mice, stained as indicated. Scale bars, 100 μm. (h) Sympathetic axon bundles isolated from subcutaneous brown adipose tissue (BAT) are enclosed by a barrier of LepR^+^ cells. Tyrosine hydroxylase (TH, green) and LepR (red) staining are shown. Scale bars, 100 μm. (i) Expression of LBP (green), LepR (red), and DRD2 (yellow) in the hypothalamus. Scale bars, 100 μm. (j, k) Micro-computed tomography images of knee joints from WT and Gdf-15 KO mice. (j) Bone Volume/Tissue Volume (BV/TV), Trabecular Thickness (Tb.Th), and Trabecular Number (Tb.N) of subchondral bone were quantified for the groups indicated above (n=6). (l, m) Representative TRAP-stained images of subchondral bone from WT and Gdf-15 KO mice, along with quantitative analysis (n=6). Scale bars, 100 μm. (n) OARSI grade of knee articular cartilage. (o) Safranin O/fast green (SO/FG) staining of knee articular cartilage. Scale bars, 100 μm. (p-w) Immunofluorescence staining of p21 (q), p53 (s), γH2AX (u), and Perilipin-1 (w) in the subchondral bone of joints from WT and Gdf-15 KO mice after restraint stress. The percentage of positive cells in the bone marrow was quantified (n=6). Scale bars, 100 μm. All data are shown as means ± SD. One way ANOVA for (b, d, j, l, n, p, r, t, v) with multiple comparisons. Student’s t test applied for (a).

We initially found a significant increase in GDF-15 levels in blood samples from CRS mice, indicating that this well-known “weight loss factor” might play a role in depression-induced cachexia, albeit arriving at an inopportune time and location (Fig. 4a). To investigate the role of GDF-15 in mediating these effects, we generated GDF-15 knockout mice (Gdf-15 KO) and tracked their weight changes under normal conditions and in the CRS model. The results showed that knockout of GDF-15 effectively mitigated the severe weight loss in depressed mice and restored thermogenesis and energy expenditure in brown adipose tissue by the end of the period (Fig. 4b-d). This was reflected in improvements in the volume of BAT (Fig. 4e), increased filling of adipose tissue (Fig. 4f), and a reduction in UCP1 expression (Fig. 4g). However, it is important to note that in the absence of depression, global Gdf-15 knockout led to excessive accumulation of adipose tissue and uncontrolled weight gain, suggesting that its benefits need to be evaluated. Corresponding to the increase in BAT volume and decreased thermogenesis, there was a significant downregulation of leptin receptors in the sympathetic nerves that innervate BAT (Fig. 4h), as well as a reduction in leptin receptor expression in the hypothalamus of brain, compared to the CRS group (Fig. 4i). Although the reduction of GDF-15 significantly improved the lean phenotype in depressed mice, we did not observe any improvement in the abnormal remodeling and destruction of the subchondral bone caused by depression. Micro-CT results still indicated osteopenia and destruction of the subchondral bone in the knee joint (Fig. 4j, k), along with excessive osteoclast activity as shown by TRAP staining (Fig. 4l, m). Additionally, there was no significant improvement in cartilage degradation and degeneration following GDF-15 knockout despite the improvement in body weight (Fig. 4n, o). Corresponding to the unaltered subchondral bone destruction, there was a significant upregulation of aging and adipogenic differentiation markers, such as p21, p53, γH2AX, and Perilipin-1, in the subchondral bone marrow (Fig. 4p-w). This finding distinguishes systemic cachexia from localized joint pathology, underscoring the need for joint-targeted therapeutic strategies.

### Osteoclast leptin receptor ablation alleviates depressive cachexia and joint pain

**Figure 5.**
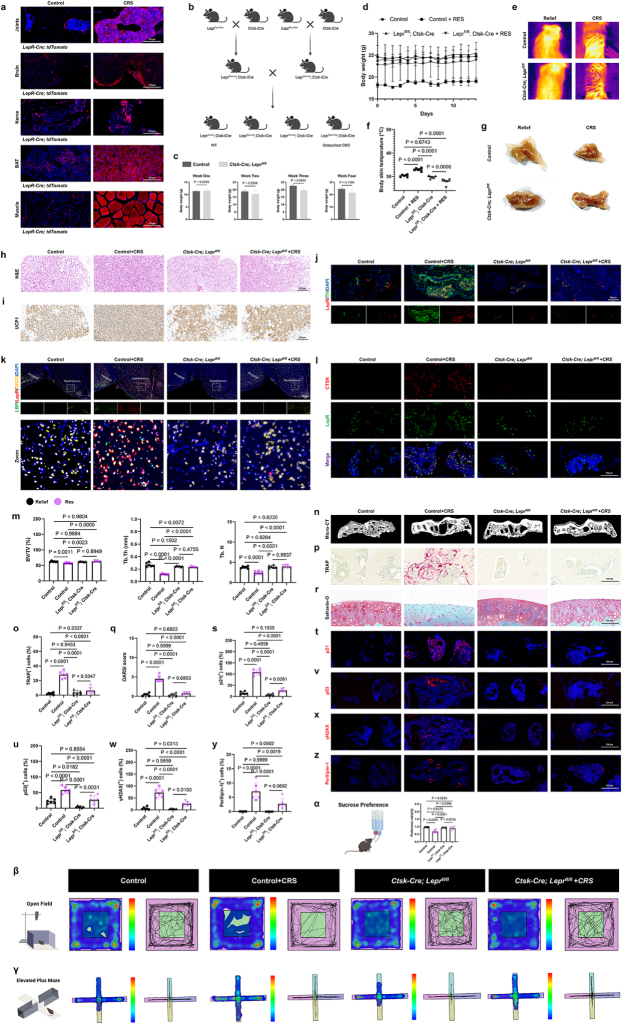
Ablation of osteoclast leptin receptor protects against depressive cachexia and joint pain. (a) Joints, brain tissue, dorsal brown adipose tissue and its innervating nerves, as well as the tibialis anterior muscle of *LepR-Cre; tdTomato mice*. (b) Schematic of mouse breeding strategy to generate conditional knockout mice lacking Lepr in osteoclasts. (c) Weekly body weight measurements of *Ctsk-Cre; Lepr^fl/fl^* mice until the fourth week (n = 3). (d) Body weight of restrained mice in Control and *Ctsk-Cre; Lepr^fl/fl^* groups (n = 4). (e, f) Thermal imaging (e) and quantification (f) at room temperature (21°C) (n = 6). (g) Representative photographs of BAT from Control and *Ctsk-Cre; Lepr^fl/fl^* mice. (h) Representative H&E-stained sections of BAT from Control and *Ctsk-Cre; Lepr^fl/fl^* mice. Scale bars, 100 μm. (i) Representative immunohistochemistry images of BAT sections from Control and *Ctsk-Cre; Lepr^fl/fl^* mice, stained as indicated. Scale bars, 100 μm. (j) Sympathetic axon bundles isolated from subcutaneous BAT are enclosed by a barrier of LepR^+^ cells. Tyrosine hydroxylase (TH, green) and LepR (red) staining are shown. Scale bars, 100 μm. (k) Expression of LBP (green), LepR (red), and DRD2 (yellow) in the hypothalamus. Scale bars, 100 μm. (l) Fluorescent co-localization of CTSK and LepR in the subchondral bone of Control mice and *Ctsk-Cre; Lepr^fl/fl^ mice*. Scale bars, 100 μm. (m, n) Micro-computed tomography images of knee joints from Control and *Ctsk-Cre; Lepr^fl/fl^* mice. (m) Bone Volume/Tissue Volume (BV/TV), Trabecular Thickness (Tb.Th), and Trabecular Number (Tb.N) of subchondral bone were quantified for the groups indicated above (n=6). (o, p) Representative TRAP-stained images of subchondral bone from Control and *Ctsk-Cre; Lepr^fl/fl^* mice, along with quantitative analysis (n=6). Scale bars, 100 μm. (q) OARSI grade of knee articular cartilage. (r) Safranin O/fast green (SO/FG) staining of knee articular cartilage. Scale bars, 100 μm. (s, t) Immunofluorescence staining of p21 (t), p53 (v), γH2AX (x), and Perilipin-1 (z) in the subchondral bone of joints from Control and *Ctsk-Cre; Lepr^fl/fl^* mice after restraint stress. The percentage of positive cells in the bone marrow was quantified (n=6). Scale bars, 100 μm. α, Sucrose preference ratio in CRS mice comparing Control and *Ctsk-Cre; Lepr^fl/fl^* groups (n=6). β, γ, Representative locomotion traces and quantitative analysis of the Open Field Test (OFT) (β) and Elevated Plus Maze (EPM) (γ) data (n=6). All data are shown as means ± SD. One way ANOVA for (d, f, m, o, q, s, u, w, y, α) with multiple comparisons. Student’s t test applied for (c).

In this part of our study, we first discovered that depressed mice also exhibited enhanced expression of *LepR-tdTomato* across multiple tissues and organs, including joints (Fig. 5a). However, the specific role of osteoclast-specific leptin receptor regulation in joint pain remains unexplored.

The breeding strategy illustrated in the diagram was designed to generate osteoclast-specific leptin receptor (Lepr) conditional knockout (CKO) mice. Lepr was floxed in mice with a *Lepr^flox/flox^* background, and these mice were crossed with *Ctsk-iCre* mice, in which the Ctsk gene promoter drives the Cre recombinase specifically in osteoclasts. The resulting offspring, *Lepr^flox/wt^; Ctsk-iCre*, were further bred with *Lepr^flox/flox^; Ctsk-iCre* mice to generate four genotypes: *Lepr^wt/wt^; Ctsk-iCre* (wild-type), *Lepr^flox/wt^; Ctsk-iCre, Lepr^flox/wt^; Ctsk-iCre*, and *Lepr^flox/flox^; Ctsk-iCre* (osteoclast-specific Lepr knockout) (Fig. 5b). After successfully generating the mouse model, we continuously recorded the body weight of the mice and confirmed that the specific knockout of the leptin receptor in osteoclasts did not affect overall body weight or development (Fig. 5c). This finding established a solid foundation for using this mouse model to investigate metabolic processes without concerns regarding potential systemic developmental disruptions. During the establishment of the CRS model, we monitored body weight for two consecutive weeks and confirmed that the knockdown of Lepr in osteoclasts successfully rescued the weight loss observed in depressed mice (Fig. 5d). Additionally, thermographic imaging of the dorsal adipose tissue revealed a similar change in energy expenditure, showing a reduction in heat dissipation that correlated with the weight-sparing effect (Fig. 5e-g). Histological analysis and immunostaining indicated that *Lepr^flox/flox^; Ctsk-Cre* adipocytes were smaller, containing more multi-locular lipid droplets, and exhibited increased expression of UCP1 (Fig. 5h, i). It is reasonable to infer that this fat burning and atrophy effect can be achieved through the regulation of sympathetic nerves innervating the adipose tissue (Fig. 5j). Finally, we also discovered that the specific knockout of leptin receptors in intramedullary osteoclasts exerts a remote regulatory effect on distal brain tissues (Fig. 5k). This further suggests that depression-related joint pain is a complex, multi-organ synergistic disease.

Next, we will delve into the changes occurring within the joints themselves. We observed that in the CRS-induced depressed mice, there was an enhancement in the expression and co-localization of CTSK and LepR in the subchondral bone, which was significantly reversed in the *Ctsk-Cre; Lepr^fl/fl^* mice (Fig. 5l). Corresponding to this reversal, we noted improvements in subchondral bone remodeling, as well as a reduction in osteoclast differentiation and degeneration of the cartilage layer (Fig. 5m-r). Notably, although the knockout of leptin receptors in osteoclasts partially improved aging and adipogenic differentiation compared to the control group, this improvement was further amplified under the conditions induced by the CRS model, affecting joint aging and adipocyte differentiation (Fig. 5s-z). Ultimately, in alignment with the regulatory effects on the joints corresponding to improvements in the brain tissues associated with depression, broader behavioral assessments further validated that targeted gene regulation aimed at the joints contributes to the improvement of depressive-like behaviors and pain (Fig. 5α-γ). Achieving overall regulation of depression with minimal joint intervention clearly holds significant clinical therapeutic value.

### Targeting subchondral bone adipogenesis relieves depression-associated cachexia and pain

**Figure 6.**
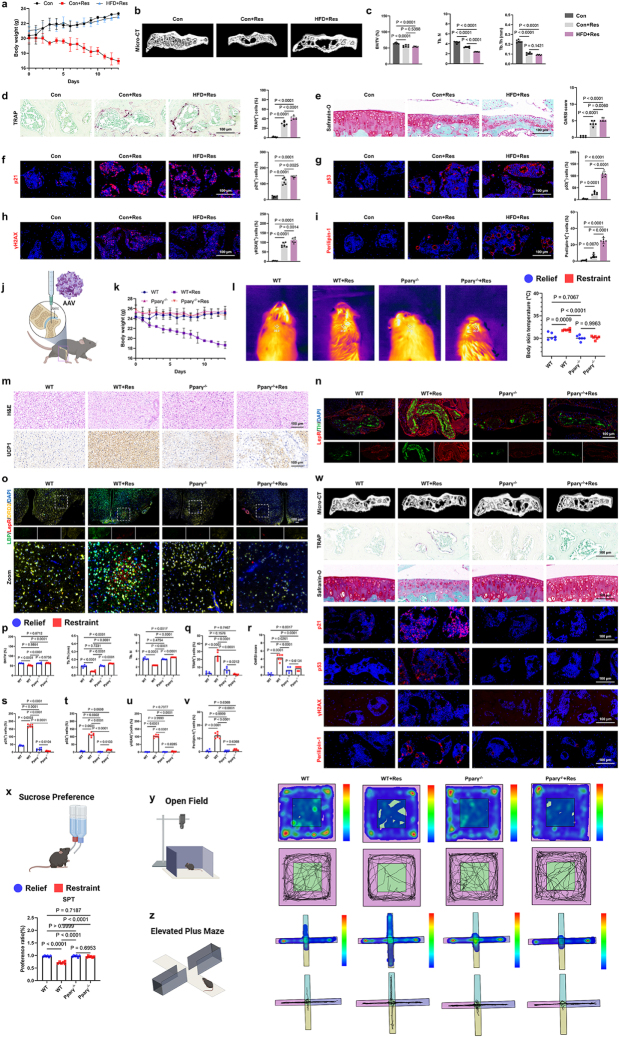
Targeting subchondral bone adipogenesis relieves depressive cachexia and pain. (a) Body weight of control and restrained mice in normal diet and high-fat diet groups (n=6). (b, c) Representative µCT images of the tibial subchondral bone, along with quantitative analysis of structural parameters (c) (n = 6). (d) TRAP staining in mouse tibial subchondral bone after restraint combined with a high-fat diet. On the right is a quantitative analysis of the number of TRAP^+^ cells per bone marrow area (mm²) (n = 6). Scale bars, 100 μm. (e) Safranin O/fast green (SO/FG) staining in mouse cartilage after restraint combined with a high-fat diet. On the right is the OARSI score (n = 6). Scale bars, 100 μm. (f-i) Immunofluorescence staining of p21 (f), p53 (g), γH2AX (h), and Perilipin-1 (i) in the subchondral bone of joints from high-fat diet mice after restraint stress. The percentage of positive cells in the bone marrow was quantified (n=6). Scale bars, 100 μm. (j) The image depicts a schematic representation of an injection of AAV (adeno-associated virus) into the tibial subchondral bone of a mouse, highlighting the structural details of the joint and the experimental model used for research purposes. (k) Body weight of control and restrained mice in WT and Pparγ KO groups (n=6). (l) Thermal imaging and quantification at room temperature (21°C) (n=6). (m) Representative H&E-stained sections and immunohistochemistry images of BAT from WT and Pparγ KO mice. Scale bars, 100 μm. (n) Sympathetic axon bundles isolated from subcutaneous brown adipose tissue (BAT) are enclosed by a barrier of LepR^+^ cells. Tyrosine hydroxylase (TH, green) and LepR (red) staining are shown. Scale bars, 100 μm. (o) Expression of LBP (green), LepR (red), and DRD2 (yellow) in the hypothalamus. Scale bars, 100 μm. (p) Bone Volume/Tissue Volume (BV/TV), Trabecular Thickness (Tb.Th), and Trabecular Number (Tb.N) of subchondral bone were quantified (n=6). (q-w) Micro-computed tomography images, representative TRAP-stained images, Safranin O/fast green (SO/FG) staining of knee articular cartilage, and immunofluorescence staining of p21, p53, γH2AX, and Perilipin-1 in the subchondral bone of joints from WT and Pparγ KO mice after restraint stress were analyzed, with the percentage of positive cells in the bone marrow quantified (n=6). Scale bars represent 100 μm. (x) Sucrose preference ratio in CRS mice comparing WT and Pparγ KO groups (n=6). (y) (z) Representative locomotion traces and quantitative analysis of the Open Field Test (OFT) (y) and Elevated Plus Maze (EPM) (z) data (n=6).All data are shown as means ± SD. One way ANOVA with multiple comparisons was used to test for significance.

We then explored whether non-genetic interventions aimed at improving body weight at a systemic level could also ameliorate depression-induced subchondral bone abnormal remodeling in mice. To achieve this experimental objective, mice in the experimental group were subjected to a high-fat diet (HFD) during the establishment of the CRS model. The depression group showed confirmed weight gain through continuous weight monitoring (Fig. 6a). However, the Micro-CT results on subchondral bone reconstruction indicated that although a high-fat diet restored the body weight of the depressed mice, it further exacerbated subchondral bone loss and damage (Fig. 6b, c). Nevertheless, both the TRAP staining results and the Safranin O/Fast Green staining results showed that a high-fat diet exacerbated osteoclast differentiation in the subchondral bone and cartilage degradation induced by the CRS model (Fig. 6d, e). The exacerbation of subchondral bone marrow adipocyte differentiation and accelerated aging induced by the high-fat diet further suggested that joint pain and cachexia resulting from depression required intervention at the joint and bone marrow level (Fig. 6f-i).

To further confirm the therapeutic value of joint-targeted interventions for systemic improvements in depression-induced cachexia, as well as for treating joint pain and remodeling, we focused on the key gene involved in bone adipogenesis—PPARγ. PPARγ knockdown using siRNAs (Small interfering RNA) was found to inhibit the rapid weight loss associated with depression (Fig. 6j, k). Thermography results indicated that local PPARγ knockdown in the bone did not have a significant impact on overall energy expenditure in mice (Fig. 6l). However, it did restore the elevated energy expenditure induced by the CRS model to normal levels (Fig. 6l). PPARγ knockdown was associated with a downregulation of UCP1 expression in adipose tissue and a rescue effect on sympathetic nerve activation and leptin receptor expression (Fig. 6m, n). Brain tissue slices from mice also showed that bone-targeted gene regulation could similarly affect brain tissue. Under the combined conditions of the CRS model, PPARγ knockdown effectively rescued the high expression of hypothalamic leptin receptors and LBP—depression-promoting proteins (Fig. 6o). This remote regulatory effect on depression and energy expenditure was not observed when PPARγ knockdown was performed in the bone alone, without the CRS model (Fig. 6o). Meanwhile, unlike the HFD, which improved mouse weight but worsened knee joint conditions (Fig. 6a-i), PPARγ knockdown in bone not only restored body weight but also effectively improved the abnormal remodeling of subchondral bone and osteoclast differentiation in depressed mice, ultimately ameliorating cartilage degradation (Fig. 6p-r and Figure 6w). Moreover, compared to the joint-related side effects observed with systemic GDF-15 knockout, PPARγ knockdown alone, without the presence of depression, did not have a significant impact on non-depressed mice (Fig. 6w). This improvement in joint conditions is likely due to the beneficial effects on bone aging and the tendency for adipocyte differentiation (Fig. 6s-v). The results from the SPT experiment revealed that genetic interventions targeting bone adipogenesis could improve depressive-like behavior in mice (Fig. 6x). Additionally, the OFT and EPM experiments further confirmed the significant regulatory value of bone aging/adipogenesis interventions in managing both depressive-like behavior and pain (Fig. 6y, z). These findings suggest a powerful potential for bone-targeted interventions to treat depression and joint pain without affecting the overall metabolic background.

### Upregulated TGF-β signaling contributes to subchondral bone architectural alterations in depression

**Figure 7.**
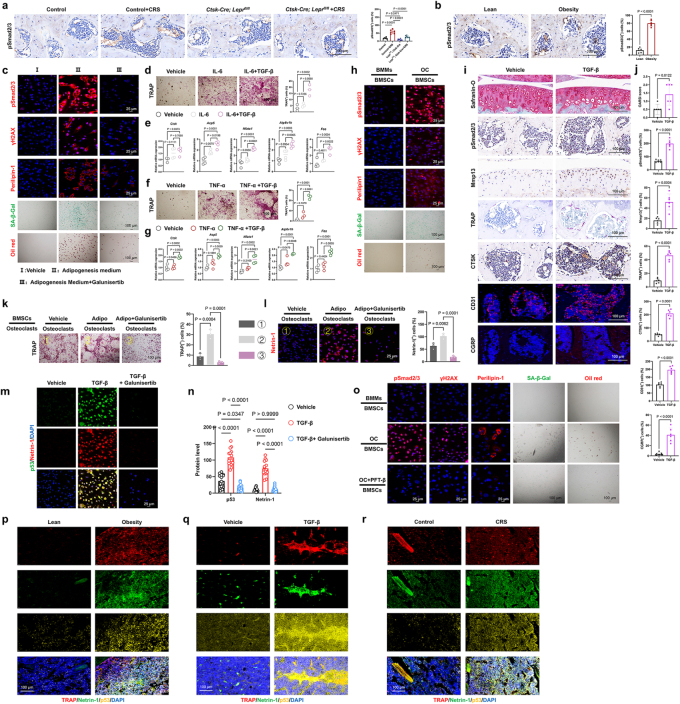
Upregulated TGF-β signaling is associated with alterations in subchondral bone architecture in depressed mice. (a) Representative immunohistochemistry images of subchondral bone from Control and *Ctsk-Cre; Lepr^fl/fl^ mice*, along with corresponding quantitative analysis, stained as indicated. Scale bars, 100 μm. (b) Representative immunohistochemistry images of subchondral bone from Lean and Obesity mice, along with corresponding quantitative analysis, stained as indicated. Scale bars, 100 μm. (c) Confocal images of immunofluorescence (IF) staining against pSMAD2/3, γH2AX, and Perilipin-1 in BMSC cells treated with vehicle, adipogenic differentiation medium, or 10 µM Galunisertib. Additionally, SA-β-Gal staining and Oil Red O staining were used to further indicate senescence and adipogenic differentiation. Scale bars, 25 and 100 μm. (d) Osteoclast differentiation determined by TRAP staining (left) and the relative area of TRAP-positive multinucleated osteoclasts per well (right) in cell cultures using bone marrow macrophages (BMMs), followed by IL-6 stimulation for 6 days in the presence or absence of TGFβ (n = 3). Scale bars, 100 μm. (e) Osteoclast differentiation determined by RT-qPCR in cell cultures using bone marrow macrophages (BMMs), followed by IL-6 stimulation for 6 days in the presence or absence of TGFβ (n = 4). (f) Osteoclast differentiation determined by TRAP staining (left) and the relative area of TRAP-positive multinucleated osteoclasts per well (right) in cell cultures using bone marrow macrophages (BMMs), followed by TNF-α stimulation for 6 days in the presence or absence of TGFβ (n = 3). Scale bars, 100 μm. (g) Osteoclast differentiation determined by RT-qPCR in cell cultures using bone marrow macrophages (BMMs), followed by TNF-α stimulation for 6 days in the presence or absence of TGFβ (n = 4). (h) Confocal images of immunofluorescence (IF) staining against pSMAD2/3, γH2AX, and Perilipin-1 in BMSC cells, with or without co-culturing with osteoclasts. Additionally, SA-β-Gal staining and Oil Red O staining were used to further indicate senescence and adipogenic differentiation. Scale bars, 25 and 100 μm. (i, j) Safranin O/Fast Green staining, TRAP staining, immunohistochemical and immunofluorescence analysis of the number of pSMAD2/3^+^, Mmp13^+^, CTSK^+^, CD31^+^, and CGRP^+^ (immunohistochemically stained brown in the images, immunofluorescently stained red in the images) cells (per mm²) in the cartilage and subchondral bone of mice (n = 6). Scale bars, 100 μm. (k) Osteoclast differentiation determined by TRAP staining (left) and the relative area of TRAP-positive multinucleated osteoclasts per well (right) in cell cultures using BMMs, with overlying BMSC cells co-cultured using adipogenic differentiation medium and 10 µM Galunisertib (n = 3). Scale bars, 100 μm. (l) Netrin-1 derived from osteoclasts determined by IF and quantitative analysis (right) in cell cultures using BMMs, with overlying BMSC cells co-cultured using adipogenic differentiation medium and 10 µM Galunisertib (n = 3). Scale bars, 100 μm. (m, n) IF staining of p53 and Netrin-1 in osteoclasts, followed by TGFβ stimulation in the presence or absence of Galunisertib (n = 15). Scale bars, 25 μm. (o) Confocal images of immunofluorescence (IF) staining against pSMAD2/3, γH2AX, and Perilipin-1 in BMSC cells, with or without co-culturing with osteoclasts, which were treated with or without PFT-β. Additionally, SA-β-Gal staining and Oil Red O staining were used to further indicate senescence and adipogenic differentiation. Scale bars, 25 and 100 μm. (p-r), Fluorescent co-localization of TRAP, Netrin-1, and p53 in the bone of obese mice and their controls, TGFβ-injected mice and their controls, as well as depressed mice and their controls. Scale bars, 100 μm. All data are shown as means ± SD. One way ANOVA for (a, d, e, f, g, k, l, n) with multiple comparisons. Student’s t test applied for (b, j).

The results showed that the elevated TGF-β expression induced by CRS in this region could be normalized in *LepR^flox/flox^; Ctsk-Cre* mice (Fig. 7a). Moreover, this upregulated TGF-β observed in depressed mice was also noted in mice on a HFD (Fig. 7b). We subsequently evaluated the regulatory effects of TGF-β on stem cell aging and adipogenic differentiation *in vitro*. To achieve this, we isolated and cultured mesenchymal stem cells from mice and utilized three culture conditions: standard α-MEM medium, adipogenic differentiation induction medium, and a combination of these with or without the classical TGF-β inhibitor, Galunisertib. The results indicated that during adipogenic differentiation, stem cells exhibited a significant upregulation of pSmad2/3 expression, along with concurrent increases in aging and adipogenesis, all of which were reversed by Galunisertib (Fig. 7c). While IL-6 and TNF-α are well-established cytokines in osteoclast differentiation^[27,28]^, our data show that using these cytokines alone, without RANKL, does not successfully induce the differentiation of pre-osteoclast BMMs into mature osteoclasts (Fig. 7d-g). However, when combined with TGF-β, multinucleated, dendritic-like osteoclasts were successfully induced, as confirmed by both TRAP staining and RT-qPCR results (Fig. 7d-g). Using a Transwell system, we co-cultured pre-osteoclasts/osteoclasts in the upper chamber and stem cells in the lower chamber^[29,30]^. In the figure (Fig. 7h), cells above the horizontal line represent those in the upper Transwell insert, while stem cells below the line correspond to those in the lower culture plate. We found that when BMMs in the upper chamber were induced to differentiate into osteoclasts using RANKL, they significantly promoted aging and adipogenic differentiation in the stem cells below (Fig. 7h). This effect was driven by the activation of TGF-β. Following the direct injection of a high dose of transforming growth factor into mice, we observed that increased TGF-β expression in the subchondral bone was accompanied by cartilage degradation, excessive osteoclast differentiation, and vascular-nerve invasion (Fig. 7i, j). These results strongly suggest that the overactivation of TGF-β disrupts the stability of the joint microenvironment.

To clarify the link between depression-induced upregulation of TGF-β, joint pain, and adipogenic differentiation, we further explored the connections between TGF-β and the joint proteins discussed earlier in this study. By switching the upper and lower layers of the Transwell co-culture system, we discovered that stem cells undergoing adipogenic differentiation in the upper chamber significantly promoted osteoclast differentiation in the lower chamber (Fig. 7k). This effect was controlled by TGF-β and was reflected in the regulation of Netrin-1 secretion by the osteoclasts in the lower chamber (Fig. 7l). These findings not only illustrate a detrimental cycle between osteoclasts and aging stem cells but also highlight the potential of TGF-β as a therapeutic target for depression-related joint pain. Applying TGF-β directly to osteoclasts resulted in an upregulation of Netrin-1 expression, consistent with cellular aging, as shown by immunofluorescence (Fig. 7m, n). Similarly, mature osteoclasts significantly accelerated aging and adipogenic differentiation in the stem cells below, just as TGF-β did when applied solely to stem cells (Fig. 7o). This effect could be reversed by inhibiting the activation of the p53 signaling pathway in osteoclasts (Fig. 7o). Ultimately, depressed mice showed increased expression of TRAP, Netrin-1, and p53, along with co-localization of bone cells, similar to findings in obese mice and those receiving direct TGF-β injections (Fig. 7p-r). This highlights the strong correlation between aging and pain in the context of depression.

### Muscle–bone crosstalk underlies depression-related joint remodeling

**Figure 8.**
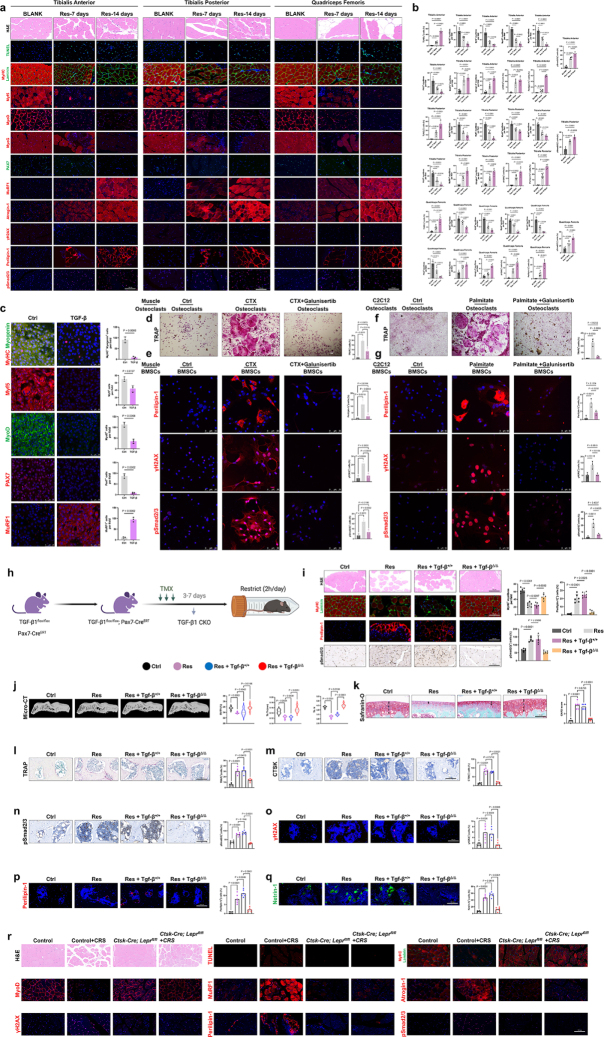
Muscle-bone crosstalk in the context of depression. (a, b) Representative images of regenerating tibialis anterior (a, left), tibialis posterior (a, middle), and quadriceps muscles (a, right) stained by hematoxylin & eosin (H&E) and immunofluorescence are shown. Corresponding fluorescent markers (a) and quantitative analysis are also presented (b) (n = 6). Scale bars, 100 μm. (c) Immunofluorescence analysis of skeletal muscle cell markers in C2C12 cells following continuous treatment with TGFβ is shown. The analysis highlights changes in marker expression levels after TGFβ exposure (n = 3). Scale bars, 50 μm. (d) Representative images showing osteoclast differentiation and co-cultured satellite cells on tibialis anterior muscle sections from Scramble- and CTS-treated mice, with or without the TGFβ inhibitor Galunisertib (n = 3). Scale bars, 100 μm. (e) Representative images of mesenchymal stem cell senescence markers, adipogenic differentiation markers, and pSMAD2/3 in co-cultured satellite cells on tibialis anterior muscle sections from Scramble- and CTS-treated mice, with or without the addition of the TGFβ inhibitor Galunisertib (n = 3). Scale bars, 50 μm. (f) Same as (d), but with the upper layer of cells in the co-culture system replaced by fatty acid-treated C2C12 cells. (g) Same as (e), but with the upper layer of cells in the co-culture system replaced by fatty acid-treated C2C12 cells. (h) Experimental design to analyze depression-induced muscle regeneration in Tgf-β^+/+^ and Tgf-β^Δ/Δ^ mice. (i) Representative images of regenerating tibialis anterior muscles stained by H&E and immunofluorescence are shown. Corresponding fluorescent markers (left) and quantitative analysis are also presented (right) (n = 6). Scale bars, 100 μm. (j-q) Micro-computed tomography images (j), representative TRAP-stained images (l), Safranin O/fast green (SO/FG) staining of knee articular cartilage (k), OARSI scoring, and immunohistochemical staining of CTSK (m) and pSMAD2/3 (n) were analyzed, along with immunofluorescence staining of γH2AX (o), Perilipin-1 (p), and Netrin-1 (q) in the subchondral bone of joints from Tgf-β^+/+^ and Tgf-β^Δ/Δ^ mice after restraint stress. The percentage of positive cells in the bone marrow was quantified (n=6). Scale bars represent 100 μm. (r) H&E staining and immunofluorescence staining of muscle regeneration markers in the tibialis anterior muscle from Control and *Ctsk-Cre; Lepr^fl/fl^* mice after restraint stress. Scale bars represent 100 μm. All data are shown as means ± SD. One way ANOVA for (b, d, e, f, g, i, j, k, l, m, n, o, p, q) with multiple comparisons. Student’s t test applied for (c).

Lastly, we also studied another crucial factor in maintaining body weight and motor function—muscle. Muscle atrophy undoubtedly plays a significant role in contributing to weight loss. Consequently, we observed a significantly increased proportion of TUNEL^+^ apoptotic cells in the tibialis anterior, tibialis posterior, and quadriceps muscles of CRS model mice compared to controls, along with a notable reduction in regenerating myofibres and the number of satellite cells (Fig. 8a, b). We also found that CRS-induced depression was accompanied by significant increases in muscle aging and adipocyte differentiation^[31]^, along with upregulation of TGF-β signaling in muscle tissue (Fig. 8a, b). This suggests that the detrimental effects of depression-induced TGF-β pathway activation may extend beyond bone tissue. Unsurprisingly, when TGF-β was applied to the C2C12 muscle satellite cell line *in vitro*, a diminished regenerative capacity of muscle fibers was observed compared to the control group (Fig. 8c).

To induce myofibre death, inflammation, and muscle regeneration, we injected cardiotoxin (CTX)^[12,32]^ into the tibialis anterior, with or without TGF-β inhibitors. After 6 days, we isolated and cultured primary muscle cells from both normal and CTX-injected muscles. These cells were then placed in the insert of a Transwell co-culture system, where they were co-cultured with osteoclast precursors and stem cells in the lower chamber. The results showed that atrophied and necrotic muscle significantly accelerated the active differentiation of osteoclasts (Fig. 8d) and promoted the aging and adipogenic differentiation of stem cells (Fig. 8e). These effects were reversed when TGF-β activation in the muscle was inhibited. To confirm that the muscle’s effects on bone metabolism were indeed significant, we also performed repeated validations using the C2C12 muscle satellite cell line, which further supported our findings (Fig. 8f, g). We also recognized that cell-based interaction studies alone were insufficient, so we conducted animal experiments to obtain more robust conclusions. To achieve this, we generated *Pax7-Cre^ERT^; TGF-β1^+/+^* and *Pax7-Cre^ERT^; TGF-β1^fl/fl^ mice* (referred to as TGF-β1^+/+^ and TGF-β1^Δ/Δ^ mice, respectively), where TGF-β1 was excised in satellite cells upon tamoxifen induction (Fig. 8h). We then confirmed that specific knockout of TGF-β in satellite cells could restore muscle regeneration impaired by depression and simultaneously improve the pronounced tendency of muscle stem cells to differentiate into adipocytes (Fig. 8i).

Micro-CT data of the animals first demonstrated that the knockdown of TGFβ in muscle satellite cells effectively alleviated depression-induced subchondral bone remodeling (Fig. 8j). Furthermore, in the absence of depression, the impact of this intervention on subchondral bone structure was not significant. The observed improvement in cartilage degeneration and the reduced OARSI score further confirmed the regulatory role of the subchondral bone microenvironment on overall cartilage metabolism (Fig. 8k). The TRAP staining and decreased CTSK expression in subchondral bone were consistent with the micro-CT findings (Fig. 8l, m). Moreover, immunofluorescence results supported the conclusion that this muscular intervention regulates subchondral bone through the TGFβ signaling pathway, influencing mesenchymal stem cell senescence and adipogenic differentiation (Fig. 8n-q). Finally, data from the experimental animals in Figure 5 demonstrated that targeted knockdown of leptin receptors in osteoclasts also improved impaired regeneration in the lower limb muscles (Fig. 8r). This suggests that the coordinated interaction between bone joints and muscles under depressive conditions, when effectively intervened, can shift from a collapsing deterioration process to a significantly improved outcome.

## Discussion

Taken together, our findings indicate that depression-induced joint pain has distinct molecular and pathological foundations, rather than being merely a psychological abnormality^[33–35]^. To date, no study has systematically elucidated how depression-induced joint pain interacts with and progresses through systemic metabolism and local remodeling. This study reports that joint pain in mouse models of depression, induced by inflammatory cytokine storms or behavioral restraint (CRS), is accompanied by organic changes in the joints. It is widely recognized that factors within society can influence adverse emotional responses such as depression and anxiety^[36,37]^. Moreover, the occurrence and development of depression induced by external stimuli are not merely a matter of psychological illness, but rather a complex pathological condition involving various physiological and biochemical reactions, as well as structural changes in the body^[38–41]^. One of the most noteworthy aspects is the involvement of inflammatory processes in the pathophysiology of depression^[42]^. The immune system regulates mood and may contribute to dysregulated inflammatory responses in depressed patients^[38,43]^. Preclinical and clinical studies postulate a key role of proinflammatory cytokines in the onset of depressive-like behaviors, as enhanced levels of cytokines, including TNF-α, IL-1β, and IL-6, have been reported in patients with major depressive disorder^[44]^. Therefore, in the study, we used the experimental method used by Fang et al. (2023)^[9]^, inducing anxiety or depression-like behavioral models in mice through intraperitoneal injection of inflammatory cytokines [lipopolysaccharide (LPS), IL-6, and TNF-α] (Fig. 1a, e, i). Changes include excessive osteoclast differentiation in the subchondral bone, vascular and neural invasion, and abnormal bone resorption and remodeling. This indicates that depressive joint pain is not merely a psychological condition in outpatient settings.

The dopamine D2 receptor mediates the action of the neurotransmitter dopamine (DA) and is crucial in the nervous system. It belongs to the G protein-coupled receptor family and is a subtype of the dopamine receptor family. D2 receptors are primarily distributed in various regions of the central nervous system, including the cerebral cortex, basal ganglia, thalamus, and hypothalamus; however, recent findings have shown their expression in the bone marrow^[23]^. Similarly, our data showed that monoamine deficiency and elevated LBP are critical factors that promote subchondral bone remodeling and joint pain in depression. TLR4 is also an essential immune receptor protein and a member of the Toll-like receptor family. As a pattern recognition receptor, TLR4 recognizes and binds to exogenous pathogen-associated molecular patterns (PAMPs), such as LPS and endogenous damage-associated molecular patterns (DAMPs), such as cellular components from tissue necrosis^[45–48]^. TLR4 is also crucial in osteoclast differentiation. When the TLR4 receptor binds to its ligand (such as bacterial LPS), downstream signaling pathways, including NF-κB and MAPK, are activated. Activation of these signaling pathways leads to the differentiation of osteoclast precursor cells into osteoclasts, promoting their fusion and activation^[49–51]^. Moreover, the upregulation of LBP effectively promotes the expression of TLR4^[9,26,52]^. We aimed to clarify whether this phenomenon also occurs in the subchondral bones of depressed mice. This study confirmed that supplementation with monoamines or regulation of the depression-related Lbp-Tlr4-Netrin-1 axis may improve subchondral bone damage and alleviate joint pain in mice. Consequently, we observed significant changes in the overall body weight and energy balance of adipose tissue in mice during the treatment of depression-induced joint pain. This suggests that depression-induced joint pain may involve the regulation and impact of systemic weight or lipid metabolism.

Enhanced aging and adipocyte differentiation within the bone marrow often indicate an imbalance in bone metabolic homeostasis and significantly contribute to diseases such as osteoporosis, fractures, and hematological dysfunction^[53–55]^. In this study, we found that while depression may lead to weight loss in mice, it exacerbates the imbalance in the bone marrow environment, resulting in bone changes similar to those observed in obesity, such as upregulation of GDF-15 and leptin receptors. We found that those bone changes are difficult to reverse through excessive nutritional supplementation alone. Fortunately, previous research suggests that targeting local lipid metabolism genes can effectively intervene in bone marrow lipid metabolism and aging without affecting overall body weight or hematological function^[54]^. Increasing evidence suggests that GDF15 plays a role in physiological appetite regulation and resistance to obesity^[56,57]^. Transgenic mice overexpressing GDF15 exhibit a lean phenotype and are resistant to obesity induced by a high-fat diet (HFD)^[58]^. Therefore, given that acutely depressed mice display a lean phenotype, we were compelled to investigate the effects of systemic changes in GDF15 expression on body weight and the deterioration of the joint microenvironment in these mice. Furthermore, by developing osteoclast-specific LepR knockout mouse models and using local injection of PPARγ knockdown viruses, we were also able to interrupt the vicious cycle between depression-induced cachexia and joint bone aging. This intervention not only halted the detrimental cycle but also successfully restored cognitive function in the experimental animals by addressing joint aging.

Research indicates that muscle and bone tissue, as well as muscle and inflammatory joint diseases, are not isolated entities but are interconnected through complex interactions. For instance, targeting muscle tissue with therapies such as Follistatin (FST), which inhibits inflammation and promotes muscle growth, could present new treatment options for obesity-related osteoarthritis^[59]^. The rich array of proteins and genetic information contained in extracellular vesicles derived from skeletal muscle can also influence the differentiation of osteoblasts and osteoclasts in bone, thereby modulating the improvement and progression of osteoporosis^[29]^. Based on the above research, we aim to clarify through *in vivo* and *in vitro* experiments whether there is an interaction between muscle and joints in the context of joint pain and weight loss induced by depression. During our exploration of regulating intra-articular fat differentiation, we uncovered the potential role of TGF-β in modulating depression-induced joint damage and weight regulation. Previously discussed, GDF-15 is a cell-stress-induced divergent member of the TGF-β superfamily^[58,60]^. TGF-β is essential for the maintenance of articular cartilage metabolic homeostasis and structural integrity^[61]^. Study has shown that inhibition of TGF-β signaling in mesenchymal stem cells of the subchondral bone alleviates osteoarthritis^[62]^. Furthermore, this factor also has significant regulatory potential on body weight^[58]^. Additionally, the previously studied leptin has also been shown to induce TGF-β synthesis through the functional leptin receptor expressed by human peritoneal mesothelial cells^[63]^. We further assessed the effect of osteoclast-specific LepR knockdown on TGF-β expression in the subchondral bone. TGF-β is also known for its anabolic effects on articular cartilage homeostasis by stimulating the production of extracellular matrix proteins and preventing the terminal differentiation of chondrocytes^[64,65]^. In this study, we found that TGF-β exacerbates subchondral bone aging and adipocyte differentiation induced by depression. Its interaction with osteoclasts leads to excessive release of the pain-inducing factor Netrin-1 and the aging factor p53. This, in turn, exacerbates depression-induced joint pain and promotes the aging and adipocyte differentiation of stem cells. The aged and adipocyte-differentiated stem cells further enhance osteoclast differentiation, creating a vicious cycle of worsening degeneration. Subsequently, the involvement of skeletal muscle surrounding the joint seems to add further complexity to the situation. Fortunately, by conditionally knocking out the overexpressed TGF-β in satellite cells, we were able to reverse the excessive muscle atrophy caused by depression and demonstrate that intervening in atrophied muscle can effectively modulate joint cartilage degeneration and subchondral bone remodeling. Conversely, by regulating adipocyte differentiation in the subchondral bone, we achieved muscle regeneration in atrophied muscles without directly affecting muscle regeneration processes. This clear interaction between muscle and joint suggests a new direction for future research: rather than focusing solely on individual tissues or specific genes, it may be more advantageous to consider gene networks across multiple sites and organs as an integrated whole.

Our findings not only elucidate the molecular basis of depression-associated joint pathology but also provide a translational framework relevant to surgical practice. Given that depression and metabolic dysregulation profoundly affect subchondral bone remodeling and pain, pre-operative screening for depressive symptoms and metabolic markers (e.g., GDF-15, leptin, lipid profiles) may help identify patients at risk of poor post-operative joint outcomes. Incorporating pre-habilitation strategies that manage depression and metabolic imbalance could further optimize surgical readiness and recovery. In addition, the identified pathways – particularly the LBP–TLR4–Netrin-1 and TGF-β signaling axes – suggest potential for localized intra-articular delivery of biologics, such as Netrin-1 neutralizing antibodies or TGF-β inhibitors, as adjuncts during arthroscopic or joint replacement procedures. Together, these insights highlight how addressing the interplay between depression, metabolism, and joint-specific remodeling can inform more comprehensive and targeted surgical interventions for degenerative joint disease.

## Limitations of the study

A limitation of the current study is that the clinical correlation in humans is based on a relatively small cohort, examining the association between depression scores and TMJOA pain or severity. While these findings support the relevance of our preclinical observations, they do not directly validate the molecular mechanisms identified in mice. Future studies with larger patient cohorts, combined with molecular analyses of joint tissue or synovial samples, will be necessary to confirm whether the pathways we identified – such as the LBP–TLR4–Netrin-1 and TGF-β signaling axes – operate similarly in humans and contribute to joint degeneration in the context of depression. Such studies could strengthen the translational significance of our findings and guide the development of targeted therapeutic strategies for patients.

## Data Availability

All data associated with this study are present in the paper or the Supplementary Materials. All materials used in the study are commercially available.
